# Tyrosyl-DNA phosphodiesterase 1 (TDP1) and SPRTN protease repair histone 3 and topoisomerase 1 DNA–protein crosslinks *in vivo*

**DOI:** 10.1098/rsob.230113

**Published:** 2023-10-04

**Authors:** Ivan Anticevic, Cecile Otten, Luka Vinkovic, Luka Jukic, Marta Popovic

**Affiliations:** Department for Marine and Environmental Research, Laboratory for Molecular Ecotoxicology, Institute Ruder Boskovic, Zagreb, Croatia

**Keywords:** DNA repair, DNA–protein crosslinks, tyrosyl-DNA phosphodiesterase 1, SPRTN, zebrafish, histones

## Abstract

DNA–protein crosslinks (DPCs) are frequent and damaging DNA lesions that affect all DNA transactions, which in turn can lead to the formation of double-strand breaks, genomic instability and cell death. At the organismal level, impaired DPC repair (DPCR) is associated with cancer, ageing and neurodegeneration. Despite the severe consequences of DPCs, little is known about the processes underlying repair pathways at the organism level. SPRTN is a protease that removes most cellular DPCs during replication, whereas tyrosyl-DNA phosphodiesterase 1 repairs one of the most abundant enzymatic DPCs, topoisomerase 1-DPC (TOP1-DPC). How these two enzymes repair DPCs at the organism level is currently unknown. We perform phylogenetic, syntenic, structural and expression analysis to compare tyrosyl-DNA phosphodiesterase 1 (TDP1) orthologues between human, mouse and zebrafish. Using the zebrafish animal model and human cells, we demonstrate that TDP1 and SPRTN repair endogenous, camptothecin- and formaldehyde-induced DPCs, including histone H3- and TOP1-DPCs. We show that resolution of H3-DNA crosslinks depends on upstream proteolysis by SPRTN and subsequent peptide removal by TDP1 in RPE1 cells and zebrafish embryos, whereas SPRTN and TDP1 function in different pathways in the repair of endogenous TOP1-DPCs and total DPCs. Furthermore, we have found increased TDP2 expression in TDP1-deficient cells and embryos. Understanding the role of TDP1 in DPCR at the cellular and organismal levels could provide an impetus for the development of new drugs and combination therapies with TOP1-DPC inducing drugs.

## Graphical abstract

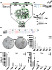

## 1. Introduction

DNA–protein crosslinks (DPCs) are very frequent DNA lesions that are triggered by byproducts of normal cellular processes such as aldehydes, reactive oxygen species and helical DNA modifications including apurinic/apyrimidinic (AP) sites [1]. They occur endogenously at a high frequency, with current estimates of 6000 lesions per cell per day [2]. DPCs can also arise from the exposure to exogenous sources such as UV light, ionizing radiation and chemotherapeutic agents [3]. These bulky lesions vary considerably depending on the type of DNA-binding protein, binding chemistry and DNA topology near the lesion (single- and double-strand breaks or intact DNA). Any protein in the vicinity of DNA can be crosslinked upon exposure to the above triggers. Histones, high mobility group (HMG) proteins and DNA-processing enzymes such as topoisomerases, DNA (cytosine-5)-methyltransferase 1 (DNMT1), poly [ADP-ribose] polymerase 1 (PARP1) and Ku70/80 have recently been identified as most common endogenous cellular DPCs [4]. Considering that DPCs affect all DNA transactions including replication, transcription, chromatin remodelling and DNA repair, the consequences of impaired repair are severe and include single- and double-strand breaks, genomic instability and apoptosis, which in turn can lead to cancer, accelerated ageing and neurodegeneration. By contrast to other DNA repair pathways that have been extensively studied for decades, DPC repair (DPCR) is a relatively new field that was only recognized as a DNA damage repair (DDR) pathway in its own right after 2014, when the first protease that initiates DPC removal from the DNA backbone was found in yeast (Wss1) [5].

DPCs can be repaired by proteolysis followed by removal of the peptide residues from the DNA backbone, or by nucleases that excise the part of the DNA to which the crosslinked protein is bound. The majority of cellular DPCs are thought to be removed by the SPRTN (Sprt-like N-terminal domain) protease in dividing cells [6–9], while the exact contribution of other proteases, including ACRC/GCNA, FAM111A, DDI1, DDI2 and proteasome, remains to be determined [10]. After proteolysis, the remaining crosslinked peptide could be repaired by the nucleotide excision repair pathway [11] or, in the case of crosslinked topoisomerases 1 and 2 (TOP1- and 2-DPCs), by tyrosyl-DNA phosphodiesterases 1 and 2 (TDP1 and 2) [12].

Topoisomerases 1 and 2 create a transient DNA–protein intermediate to reduce DNA torsional stress. Camptothecin (CPT) and its derivatives are used in cancer therapy to stabilize TOP1 intermediates and prevent replication of cancer cells [13]. TDP1 is a critical enzyme for TOP1-DPCR and is a promising target for cancer treatment [14,15]. The phosphotyrosyl bond between TOP1 and DNA is hydrolyzed by TDP1, releasing the TOP1 residue from the DNA backbone [16]. This bond is normally shielded and becomes accessible to TDP1 after partial proteolysis of TOP1. However, the dominant protease acting upstream of TDP1 has not yet been identified. While SPRTN has previously been considered the major protease for TOP1-DPCR [9,17,18], other proteases such as FAM111A, DDI1 and DDI2, as well as the proteasome, have also been associated with TOP1-DPC proteolysis [19–21]. Alternatively, another mechanism involving the 3′-flap endonucleases (Mus81/Eme1, Mre11/Rad50 and XPF/ERCC1) can facilitate TOP1-DPC removal without the need for proteolysis [15,22–25]. However, the nucleolytic pathway may not be as effective in cancer cells which often have mutated or inactivated DNA checkpoints required for endonuclease activation [26], potentially making them more dependent on repair mediated by proteases and TDP1. Mutations in TDP1 and SPRTN lead to the neurological disorder spinocerebellar ataxia with axonal neuropathy-1 (SCAN1) [27] and Ruijs–Aalfs syndrome (RJALS) characterized by premature ageing and early onset liver cancer [28], respectively.

In addition to tyrosyl-3′-phosphodiester crosslink, TDP1 can resolve a variety of substrates at the 3′ end of DNA [29,30]. Recently, it was shown that TDP1 can remove histones H2B and H4 attached to the AP sites in the *in vitro* system [31] without the need for prior proteolysis. However, TDP1 is unable to remove larger DPCs including Parp1 and TOP1 *in vitro* [31–33]. It is currently unknown how these discoveries translate to the *in vivo* system. DPC analysis was not performed in the *Tdp1*^−/−^ mouse model or in human-derived SCAN1 patient cells, because the syndromes and models were characterized long before the characterization of the DPCR pathway as a separate DNA damage pathway in 2014 and 2016 [15,27]. Understanding the role of TDP1 in DPCR at the cellular and organismal levels could provide an impetus for the development of new drugs and combination therapies with TOP1-DPC inducing drugs. Therefore, we have set out to investigate whether TDP1 can repair histone and TOP1-DPCs in cells and in an animal model and whether upstream proteolysis by SPRTN is required. Previously, it was thought that TDP1 specifically repairs TOP1-DPC remnants in cells, whereas data at the organism level were lacking. Our model organism, zebrafish (*Danio rerio*), is a well-established vertebrate system used to study cancer and neurodegenerative and cardiovascular diseases [34,35]. In zebrafish, DNA damage genes and pathways are 99% conserved compared to humans. Unlike mice, zebrafish are externally fertilized, which significantly facilitates gene editing, embryo manipulation and DPC analysis. In addition, the very high fecundity allows for better statistical analysis of DPC formation and repair in both embryos and adults [35,36].

In this study, we investigate the function of TDP1 and SPRTN in the repair of TOP1- and histone H3-DPCs, as well as in the repair of total cellular DPCs in human cells and in a zebrafish model. To this end, we compare human, mouse and zebrafish TDP1 orthologues using phylogenetic, syntenic, structural and expression analysis and show that zebrafish is a suitable vertebrate model to study TDP1 function. We combine knockdown of *sprtn* with a Tdp1-deficient zebrafish model and RPE1 cell lines with reduced expression of *TDP1* and *SPRTN*. For this purpose, we developed and verified a new set of tools, including a Tdp1-deficient zebrafish strain, a zebrafish Tdp1 antibody, morpholino probes for *sprtn* silencing in embryos, a modification of the RADAR (rapid approach to DNA adduct recovery) assay for DPC isolation from tissues, and detection of TOP1- and H3-DPCs in embryonic tissues. We show that TDP1 and SPRTN are required for *in vivo* resolution of TOP1 and H3 DPCs and that both enzymes are also required for repair of other cellular DPCs. By contrast to H3-DPCR, where SPRTN and TDP1 function together, we show that they work in separate pathways during repair of endogenous TOP1-DPCs. Our results reveal the relationship between TDP1 and SPRTN in the repair of DPCs in human cells and in an animal model, providing the first in-depth insights into the repair of DPCs at the organism level.

## 2. Material and methods

### 2.1. Zebrafish husbandry and chemical treatment of embryos

Zebrafish (*D. rerio*) AB were purchased from the European Zebrafish Resource Centre (EZRC, Karlsruhe, Germany). Fish were maintained at a constant temperature of 28°C and on a 14 h light and 10 h dark cycle, with water quality (temperature, pH and conductivity) monitored daily. Embryos were maintained in E3 media (5 mM NaCl, 0.17 mM KCL, 0.33 mM CaCl_2_ and 0.33 mM MgSO_4_) in petri dishes in an incubator at 28°C until 2 days postfertilization (dpf). Two-day-old embryos were dechorionated and treated with 10 µM CPT (Alpha Aesar: J62523) for 1 h or 5 mM formaldehyde (FA, KEMIKA: 0633501) for 30 min. All handling and experiments were performed in accordance with the directions given in the EU Guide for the Care and Use of Laboratory Animals, Council Directive (86/609/EEC) and the Croatian Federal Act on the Protection of Animals (NN 135/06 and 37/13) under the project licence HR-POK-023.

### 2.2. Cell culture, siRNA and chemical treatments

HEK293T (human embryonic kidney cells) and RPE1 (retinal pigmentosum epithelial cells 1) cell lines were used in this study. Cells were cultured at 37°C in Dulbecco's modified Eagle's medium (Capricorn, DMEM-HPA) containing 10% FBS (Capricorn, FBS-11A) serum under 5% CO_2_. RPE1 cells were seeded at 1 × 10^4^ cells ml^−1^ in 75 cm^2^ cell culture plates. The siRNAs targeting *TDP1*, *TDP2* and *SPRTN* and a control siRNA (siCTRL) (table 1) were transfected with Dharmafect reagent (Horizon Discovery, T-2005-01) according to the manufacturer's instructions. Cells were incubated for 72 h; they grew to 80–100% confluence and were collected for the RADAR assay and qPCR experiments. Silencing efficiency was determined by qPCR using the primers listed in table 5. Before collection, cells were treated with 50 nM CPT for 1 h in serum-free media or incubated with 1 mM FA for 20 min in ice-cold serum-free media.

**Table 1.** siRNAs used for gene silencing in RPE1 cells, with corresponding concentrations, manufacturers and catalogue numbers.

siRNAs	sequence	*c* (nM)	source
HsTDP1-1	5′CACAAAUGGUCAGCUGAGA3′	25	Sigma-Aldrich (PDSIRNA2D)
HsTDP1-2	5′CGAUGAAUCAAAGUGGUUA3′	10	Horizon Discovery (E-016112-00-0005)
	5′GGACCAGUUUAGAAGGAUA3′		
	5′CUGGGGUGUUGUAUGUAUU3′		
	5′GCUAAGGCCUAGAAGGUUA3′		
HsTDP2-1	5′GCCAAGAGAUUAUUCCUUU3′	5	Horizon Discovery (D-017578-02-005)
HsTDP2-2	5′GCAAGAGGCUCCAGAGUCA3′	5	Horizon Discovery (D-017578-01-005)
HsSPRTN-1	5′CAUCAAAGUCAAAAGCGAA3′	5	Horizon Discovery (L-015442-02-0005)
HsSPRTN-2	5′CAAGGAUAAGUGUAACAGUTT3′	5	ThermoFisher (4392420)
siCTRL	5′AAGUGGAGCGUGCGAAUGA3′	10	Santa Cruz Biotechnology (sc-37007)

**Table 5.** List of primers used for human (Hs), zebrafish (Dr) and mouse (Mm) qPCR analysis. Primers were purchased from Macrogen (Europe).

oligonucleotide name	sequence
HsTDP1-F	5′GGGACGCTTGTTTCTTCAGC3′
HsTDP1-R	5′TCACCATGCACAAGCAGGAT3′
HsSPRTN-F	5′GAGGTGGATGAGTATCGGCG3′
HsSPRTN-R	5′GGGTTCCCTGTTAGTAGCTCG3′
HsTDP2-F	5′CCAGTATACATGGGATACACAAATG3′
HsTDP2-R	5′TCTGCTGCTGCTCTGAAAAATA3′
HsATP50-F	5′ATTGAAGGTCGCTATGCCACAG3′
HsATP50-R	5′AACAGAAGCAGCCACTTTGGG3′
DrTdp1-F	5′ACAGATGCTCCTGATTTACCCA3′
DrTdp1-R	5′TGTGCCGTCTGTATGCTGTA3′
DrSprtn-F	5′AATGACAAGTTCTTCTGGGGG3′
DrSprtn-R	5′AAACACCAGCACATAGCGTCA3′
DrTdp2a-F	5′CAGAGTCTCTCCAATGTCAATCCA3′
DrTdp2a-R	5′TGGGTGCACTTGGTTTCTGT3′
DrTdp2b-F	5′ATGGATTCAGTCTTCGATGAGG3′
DrTdp2b-R	5′CTGTCAAGTCAATGCAATCCGC3′
DrAtp50-F	5′CTTGCAGAGCTGAAAGTGGC3′
DrAtp50-R	5′ACCACCAAGGATTGAGGCAT3′
MmTdp1-F	5′TTGGAACACACCACACGAAA3′
MmTdp1-R	5′GGGTTTTCTGGTGCCAGTCT3′
MmAtp50-F	5′TATGCAACCGCCCTGTACTC3′
MmAtp50-R	5′CCTTCAGGAGTTGCCCTACG3′
Mm18SrRNA-F	5′GATGGTAGTCGCCGTGCCTA3′
Mm18SrRNA-R	5′CCTGCTGCCTTCCTTGGA3′

### 2.3. Phylogenetic and syntenic analyses and structural modelling

Blast searches were performed using the NCBI database (National Center for Biotechnology Information) and human TDP1 sequence across bacteria, yeast, algae, plants, fungi, invertebrate and chordate species. Full length protein sequences were aligned using the MAFFT (multiple alignment using fast Fourier transform) alignment algorithm [37]. Alignment quality score was assessed using the Guidance2 server and was 0.563, indicating sufficiently high alignment quality for tree building [38]. The phylogenetic tree was constructed using the maximum-likelihood method (PhyML software with optimized tree topology, LG model, eight rates of categories, tree searching operation best of NNI&SPR (nearest neighbour interchange and subtree pruning and regrafting)) [39]. Branch support Alrt values (approximate likelihood-ratio test) are shown at tree nodes on a scale of 0–1, where 1 is maximum node confidence [40]. Synteny *tdp1* gene analysis between zebrafish, human and mouse were performed using Genomicus, a browser for conserved synteny synchronized with genomes from the Ensembl database [41]. Zebrafish Tdp1 was modelled using the Phyre2 workspace [42] and human TDP1 (PDB: c1nopB) as a template. The degree of protein disorder was predicted using PONDR-FIT software [43].

### 2.4. Generation of the *tdp1* mutant zebrafish line

**Figure 1.**
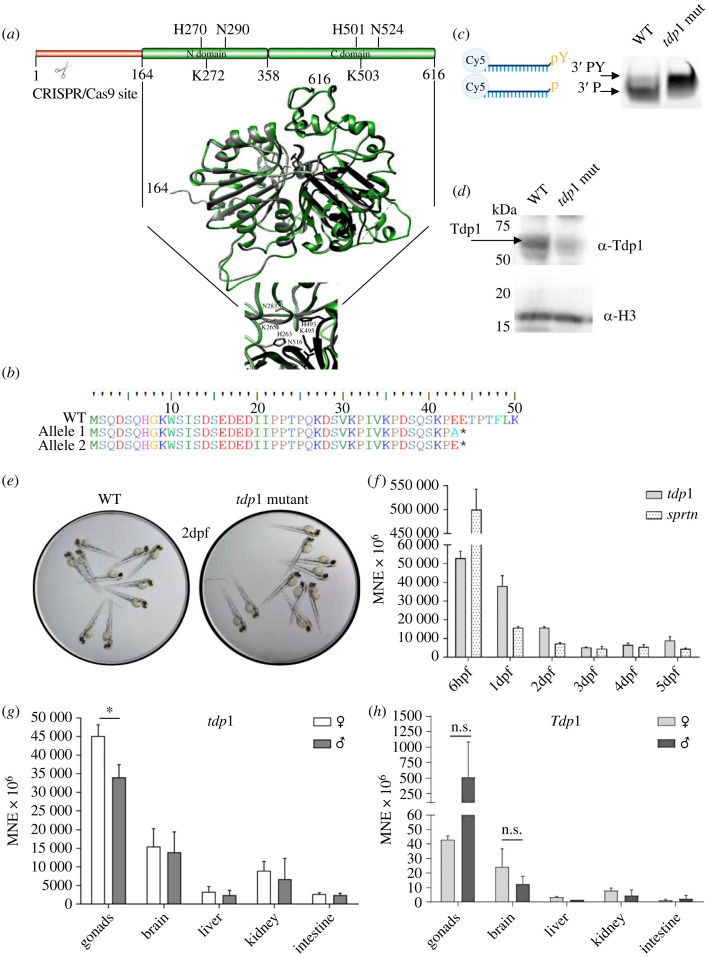
Structural comparison of zebrafish and human TDP1, validation and characterization of the zebrafish *tdp1* mutant line, and *tdp1* expression profiles in zebrafish embryos and zebrafish and mouse tissues. (*a*) The zebrafish Tdp1 structural model (in green) is overlapped with the human TDP1 crystal structure (PDB: 1jy1) [44], shown in grey (N domain) and black (C domain). Zebrafish Tdp1 was modelled using the Phyre2 workspace [42] according to the human TDP1 (PDB: c1nopB). N domain and C domain form a pseudo-2-fold axis of symmetry where each domain contributes to the active site: H263, K265 and N283 in the N domain and H493, K495 and N516 in the C domain. (*b*) Amino acid sequence of Tdp1 in *tdp1* mutant fish line: frameshift and introduction of a premature stop codon in *tdp1* mutant fish line is deduced from DNA sequencing (*, premature STOP). (*c*) TDP1 activity assay performed with 600 ng of lysate from 2-dpf WT and *tdp1* mutant embryos. Left panel: scheme created with BioRender.com of TDP1 substrate oligonucleotide with tyrosine (pY) on 3′ end and Cy5 fluorescent reporter on 5′ end and a reaction product after TDP1-mediated removal of tyrosine (p); right panel: TDP1 activity assay reactions resolved on 20% homemade urea gel and visualized using the ChemiDoc MP Imaging System to detect Cy5 fluorescence. (*d*) Western blot using a custom antibody against zebrafish Tdp1 shows the absence of a specific Tdp1 signal (68 kDa, indicated by arrow) in *tdp1* mutant embryo lysate. Histone H3 was used as a loading control. (*e*) Images of WT and *tdp1* mutant embryos (2 dpf, 2 days post fertilization). Embryos were maintained in E3 media, placed on a lid of a 96-well culture plate, and visualized with stereo microscope (Motic-SMZ-171-TP). Images were captured using a Canon 250D DSLR camera. (*f*) *Tdp1* and *sprtn* expression patterns during the embryonic development from 6 h post fertilization (6 hpf) to 5 days post fertilization (5 dpf). Data represent MNE (mean normalized expression) ± s.d. (*n* = 3) normalized to the housekeeping gene *atp50*. (*g*) Tissue expression pattern of *tdp1* in male and female zebrafish, with statistically significant differences between expression in ovaries and testes (*p < 0.05) determined by unpaired *t*-test. Data are presented as MNE (mean normalized expression) ± s.d. (*n* = 3) normalized to the housekeeping gene *atp50.* (*h*) Tissue expression pattern of *Tdp1* in male and female mice (n.s., non significant, *p* > 0.05). Data represent MNE (mean normalized expression) ± s.d. (*n* = 3) normalized to the housekeeping gene *Atp50*.

The short guide sgRNA (5′GAATGTGGGGGTCTCTTC3′) targeting exon 2 (figure 1*a*) was selected using the CRISPR scan algorithm [45] and was generated as previously described [46]. A mixture containing 600 ng µl^−1^ of Cas9 protein (NEB: M0386) and sgRNA complex at a 1 : 1 ratio was generated with a total volume of 3 µl, and 1 nl was injected into one cell stage embryos. Three months after injection, when the F0 zebrafish reached adulthood, they were outcrossed to WT, and their progeny was analysed using high-resolution melting analysis (HRMA) and sequencing to identify founder fish carrying the target mutation with the primers shown in table 2. Founder fish were crossed and the F1 generation was raised and genotyped. Female and male were found to have a premature stop codon mutation (electronic supplementary material, figure S1*e*), and they were bred to produce the F2 generation harbouring two changes in exon 2 of the *tdp1* gene.

**Table 2.** List of oligonucleotides used for the *tdp1* zebrafish mutant line creation and genotyping. All oligos were purchased from Macrogen (Europe).

oligonucleotide name	sequence	purpose
IVT-T7-F	5′GGATCCTAATACGACTCACTATAG3′	guide creation
OLIGO-SCAFF-R	5′AAAAAAGCACCGACTCGG3′	guide creation
SCAFFOLD	5′AAAAAAGCACCGACTCGGTGCCACTTTTTCAAGTTGATAACGGACTAGCCTTATTTTAACTTGCTATTTCTAGCTCTAAAAC 3′	guide creation
DrTdp1-guide	5′GGATCCTAATACGACTCACTATAGGAATGTGGGGGTCTCTTCGTTTTAGAGCTAGAA3′	guide creation
DrTdp1-genotype-F	5′GTGAAACCAGATTCGCAAAGCA3′	genotyping
DrTdp1-genotype-R	5′GTTTTGGACTCAGTCTGGGCT3′	genotyping

### 2.5. sgRNA synthesis and microinjecting procedure

To generate a *tdp1* mutant line, we targeted the first exons of zebrafish Tdp1, and the CRISPR scanning algorithm selected (5′GAATGTGGGGGTCTCTTC3′) as the sgRNA with the highest score of 54 and a low off-target effect of 4.63 CFD [45]. The gRNA was generated according to the established protocol of [46]. Briefly, the sgRNA template was inserted between two short DNA sequences, one containing the T7 promoter and the second sequence complementary to the scaffold template. In the PCR mixture, we combined the guide DNA template for the sgRNA, the IVT-T7-F primer that anneals at the beginning of the template (T7 promoter), the scaffold template and the OLIGO-SCAFF-R primer that sits at the end of the scaffold sequence (table 2). The PCR product serves as the template for *in vitro* T7 transcription using the MEGAshortscript T7 Transcription Kit (Invitrogen, AM1354). *In vitro* transcription was performed according to the manufacturer's instructions, and the transcribed RNA was purified using the Monarch RNA Cleanup Kit (NEB, T2040L), RNA concentration was measured, and the guide RNA was stored at −80°C. On the day of injection, guide RNA was incubated with Cas9 protein in a 1 : 1 ratio for 5 min at 37°C. The sgRNA/Cas9 complex was pipetted into a Femtotips needle (Eppendorf, 5242957000) and connected to a microinjector (Eppendorf, FemtoJet 4x).

### 2.6. Tdp1 activity assay

The activity assay was performed as described in [47]. Briefly, embryo lysates were incubated with a TDP1 oligonucleotide substrate (5′GATCTAAAAGACT3′) containing a tyrosine at the 3′ DNA end and Cy5 at the 5′ end for visualization, which was purchased from Midland Certified Reagent Company. If TDP1 is active, a shift in the size of the oligonucleotide substrate becomes visible, revealing the removal of the tyrosine from the substrate. In brief, 2-day-old mutants and WT embryos were deyolked in ice-cold PBS, and pelleted embryos were homogenized twice for 10 s (Ultra Turrax T25, IKA-Janke and Kunkel) in 200 µl of lysis buffer (200 mM Hepes, 40 mM NaCl, 2 mM MgCl_2_, 0.5% Triton X-100 with protease inhibitors (leupeptin, aprotinin, chymostatin, pepstatin at a concentration of 1 µg ml^−1^ and PMSF at a concentration of 1 mM)) and incubated on ice for 30 min. The supernatant protein solution (600 ng) was incubated with 2.5 µM labelled oligonucleotide substrate (Midland Certified Reagent Company) in assay activity buffer (25 mM Hepes (pH 8.0), 130 mM KCl and 1 mM dithiothreitol (DTT)) to a final reaction mixture of 10 µl. The reaction was incubated at 37°C for 1 h, then loading buffer was added, followed by boiling at 90°C for 10 min. All samples were loaded onto a pre-run 20% homemade urea gel and run at constant voltage (150 V) for approximately 1 h. The oligonucleotide substrate products were visualized using the ChemiDoc MP Imaging System to detect Cy-5 fluorescence (Bio-Rad, 1708280).

### 2.7. Zebrafish gene silencing with morpholino oligonucleotides

Two morpholino oligonucleotides, one targeting the 5'-UTR region of the zebrafish *sprtn* gene and the other targeting the exon 2-intron 2 boundary, were designed to block zebrafish *sprtn* transcription, ordered from Genetools (USA), and used as described in [48] (table 3). One nanolitre of the microinjection mixture containing 250 µM of each MO, 0.3 M KCl and 0.015% phenol red was injected into each one-cell stage embryo. Two-day-old morphants were collected as follows: (i) 5 embryos for qPCR analysis and to check the efficiency of morpholino splice blocking, (ii) 30 embryos for RADAR experiments. Total RNA was extracted using the Monarch Total RNA Kit (NEB, T2040) according to the manufacturer's instructions. Equal amounts of RNA were reverse transcribed using the High-Capacity cDNA Kit (Applied Biosystems, 4368814). The splice-blocking efficiency of morpholino was verified by PCR on 5 ng of cDNA with primer pairs annealing upstream and downstream of the morpholino target (table 4): the 439 bp amplicon from WT and the 339 bp amplicon from *sprtn* morphants caused by exon 2 skipping were separated by 1% agarose gel electrophoresis. Silencing efficiency was quantified using ImageJ (electronic supplementary material, figure S2*a*,*b*). The same cDNA was used for qPCR analysis with the primers listed in table 5.

**Table 3.** List of morpholinos used to silence *sprtn* expression in zebrafish. The morpholinos were obtained from Genotools (USA).

morpholino	sequence	target
DrSprtn-Mo-1	5′TCGGTCTGCTTTAGTAACAACAGTT3′	5′UTR
DrSprtn-Mo-2	5′AGAGAGGCATATTTAACCAACCTGA3′	ex2-in2 boundary

**Table 4.** Primers used to determine the silencing efficiency of the exon skipping morpholino. The primers were obtained from Macrogen (Europe).

primer	sequence
DrSprtn-Mo-F	5′ACTGTCCGTCCAGTAAGAGG3′
DrSprtn-Mo-R	5′CCACTTGCTTGGTTGATTCTGT3′

### 2.8. DPC isolation, detection and quantification

DPC isolation and detection was performed using the modified RADAR (rapid approach to DNA adduct recovery) assay [49]. We modified the RADAR assay in order to increase processivity and reduce variability between experiments. We also optimized DPC isolation from zebrafish embryos (1–3 dpf). The main modification included the use of a lyophilizer which substituted the TCA precipitation step, thus significantly reducing sample loss and variability between samples. DPC isolation from cells was performed with 8 × 10^6^ cells grown in a 75 cm^2^ cell flask. DPC isolation from 2 dpf embryos was performed as follows: (i) lysis of 30 embryos per condition with pre-warmed lysis buffer (6 M GTC, 10 mM Tris–HCl (pH 6), 20 mM EDTA, 4% Triton X100, 1% *N*-lauroylsarcosine sodium and 1% β-mercaptoethanol) for 10 min at 50°C; (ii) precipitation of DNA with crosslinked proteins by adding an equal volume of 98% ethanol; (iii) centrifugation at 10 000 rcf for 10 min at 4°C; (iv) washing the pellet four times with wash buffer (20 mM Tris (pH 7.4), 1 mM EDTA, 50 mM NaCl, 50% EtOH) with centrifugation steps at 10 000 rcf at 4°C; (v) dissolving the pellet in 8 mM NaOH. A 25 µl aliquot of the DPC sample was treated with proteinase K (15 µg, Fisher Scientific, BP1700-100), and the DNA was quantified using Pico Green according to the manufacturer's instructions (Invitrogen, P7581). The DPC samples were normalized to the one with the lowest amount of DNA and treated with DNAse (Millipore, E1014) for 1 h at 37°C. They were then snap-frozen in liquid nitrogen, submitted to overnight lyophilization, and then dissolved in SDS loading buffer (4 M urea, 62.5 mM Tris–HCl (pH 6.8), 1 mM EDTA, 2% SDS). Lyophilization step included freeze-drying at −48°C (5 Pa vacuum) overnight using a FreeZone 2.5 lyophilizer (Labconco, USA). Total DPCs were resolved by SDS–PAGE gradient gel (5–18%) and detected by silver staining according to the manufacturer's protocol (Sigma Aldrich, PROTSIL1). For specific DPC detection, DPCs isolated from cells were applied to the nitrocellulose membrane (GE Healthcare, 10-6000-02) for dot blot analysis, while DPCs isolated from embryos were first resolved by SDS-acrylamide gradient gel (5–18%), transferred to PDF membrane (Roche, 03010040001) and then visualized by western blotting. To detect the presence of histone H3 or TOP1 in the DPCs, the membranes were immunostained with the specific antibodies. For detection of histone H3, 200 ng of DNA-normalized DPCs was subjected to dot blot or western blotting, and then immunostained with anti-H3 antibody (Cell Signaling, no. 9715, 1 : 3000). For detection of TOP1, DPC equivalent of 500 ng DNA was applied to a dot blot (Bethyl, A302-589A, 1 : 1000), and an equivalent of 1 µg of DNA-normalized DPCs was resolved using SDS-acrylamide gradient gel with the addition of 4 M urea (Cell Signaling, no. 38650, 1 : 1000). DNA detection was performed by applying 2 ng of the sample collected for Pico Green detection to a nylon membrane and detecting with the α-dsDNA antibody (abcam ab27156, 1 : 7000).

### 2.9. MTT viability test

The MTT colorimetric assay was used as an indicator of cell viability as previously described [50]. In brief, RPE1 cells were seeded in 24-well plates and transfected with siRNA. After 72 h, cells were incubated with 100 µl of a 5 mg ml^−1^ MTT solution (Alfa Aesar, L11939) for 3 h. The solution was removed and 500 µl isopropanol (Kemika, 1622601) was added, followed by shaking at 350 rpm for 15 min (BioSan, PST-60HL-4, Plate Shaker-Thermostat). Absorbance was measured at 570 nm using a microplate reader (Tecan, Infinite M200).

### 2.10. Quantitative PCR analysis

Zebrafish (AB strain, eight months old) were anaesthetized by overdose of tricaine methane sulfonate (MS222, 200 mg l^−1^) by prolonged immersion (10 min). Tissue specimens of three animals were pooled together for the qPCR analysis due to small organ size as previously described [51]. To ensure minimal RNA degradation, organs were carefully dissected on ice, and all surfaces and equipment were thoroughly cleaned with RNAse away solution. Frozen mouse tissues from single individuals of both genders (129S1/SvImJ WT, Stock No. 002448, four months old) were a kind gift of dr.sc. Tihomir Balog (Ruder Boskovic Institute, Croatia). Tissues from three females and three males were analysed. The tissues were mechanically homogenized using an Ultra Turrax T25 homogenizer at medium intensity for 60 s, followed by incubation with proteinase K at 55°C for 5 min. Subsequently, the samples were centrifuged at 16 000 × *g* for 2 min at room temperature, and the supernatant containing the RNA was collected. RNA isolation was performed using the Monarch Total RNA Miniprep Kit (NEB, T2040L). For reverse transcription, the ProtoScript II First Strand cDNA Synthesis Kit (NEB, E6560L) was used. Total RNA from zebrafish or mouse tissues was added in a volume corresponding to 1000 ng of RNA, resulting in a concentration of 50 ng µl^−1^ of cDNA for subsequent qPCR analysis. qPCR analysis was performed with the GoTAQ qPCR mix (Promega, A6001) using primer pairs that span exon–exon boundaries to exclude possible amplification from genomic DNA (table 5). Target gene expression was normalized to a housekeeping gene, the *ATP50* gene [52], which is similarly expressed in all samples (embryonic developmental stages and adult tissues, data not shown). Quantification was performed using the Q-gene [53] method and gene expression is expressed as MNE (mean normalized expression) where $\mathrm{MNE}=E(\mathrm{HKG}{)}^{\mathrm{Ct}(\mathrm{HKG})}/E(\mathrm{gene}{)}^{\mathrm{Ct}(\mathrm{gene})}\times 10^{6}$. *E* (HKG) is the primer efficiency of the housekeeping gene; *E* (gene) is the primer efficiency of the target gene; Ct (HKG) is the mean Ct value for the housekeeping gene; and Ct (gene) is the mean Ct value of the target gene. The expression level of the target gene was then normalized to the housekeeping gene *ATP50* in zebrafish and 18S rRNA in mouse.

### 2.11. High-resolution melting curve analysis

High-resolution melting curve analysis was used to distinguish mutants from WT fish based on differences in dsDNA melting fluorescence. The primer pair listed in table 2 (DrTdp1-genotype), along with genomic DNA and MeltDoctor HRM Mastermix (Applied Biosystems, 4415440) were added to a 10 µl PCR mix, and fluorescence was detected using the StepOnePlus Real-Time PCR System (Applied Biosystems, 4376600). Data were analysed using high-resolution melting (HRM) software and melting curve changes were confirmed by sequencing.

### 2.12. Development and verification of a custom-made zebrafish Tdp1 antibody

The peptide N-GALEKNNTQIMVRSYE-C was specifically chosen to detect both zebrafish and human TDP1 proteins and was used to immunize two rabbits followed by affinity purification of serum (Genosphere, UK). To test the specificity of the antibody, we performed western blotting on WT and *tdp1* mutant zebrafish samples, as well as on WT HEK293T cells and HEK293T cells overexpressing human TDP1. Cells were transfected with the recombinant plasmid carrying the human *TDP1* gene (GenScript, OHU22350D) using PEI transfection reagent as previously described [54,55]. Cells were collected after 72 h and lysed in RIPA buffer (150 mM NaCl, 1% Triton X-100, 0.50% Na-deoxycholate, 0.10% SDS, 50 mM Tris–HCl (pH 8)) followed by a 10 s sonication; 2 dpf zebrafish embryos were lysed in RIPA buffer and sonicated twice for 10 s. Thirty micrograms of protein extract from cells and 50 µg from zebrafish embryos were loaded on an SDS acrylamide gel, transferred to a PVDF membrane, blocked with milk for 2 h, and incubated overnight with the custom-made anti-Tdp1 antibody (1 : 1000). The blot was visualized by incubating the membrane with an HRP-labelled anti-rabbit antibody followed by detection using ECL (Biorad) at the Chemidoc (Biorad).

### 2.13. Western blotting and dot blotting

Zebrafish tissues (AB strain, 10 months old, pool of three organs) were submerged in RIPA lysis buffer (150 mM NaCl, 0.1% SDS, 1% Triton X-100, 0.50% Na-deoxycholate, 50 mM Tris–HCl, pH 8) supplemented with protease inhibitors (leupeptin, aprotinin, chymostatin, pepstatin at a concentration of 5 µg ml^−1^ and PMSF at a concentration of 5 mM) and kept on ice. Tissues were homogenized using an Ultra Turrax T25 homogenizer (3 × 20 s). After homogenization, SDS was added at a final concentration of 0.8%, and the tissues were incubated on ice for 15 min. Samples were then centrifuged at 8000*g* for 10 min at 4°C, and the supernatant was collected, aliquoted and stored at −80°C. Protein concentration was determined using the Bradford assay [56]. Protein samples were analysed by western blot (50 µg per well). Immunoblotting was performed with a custom-made zebrafish Tdp1 antibody at a dilution of 1 : 1000, while anti-H3 antibody (Cell Signaling, no. 9715, 1 : 3000) was used as loading control.

The cell/embryo lysates or isolated DPCs were mixed with 5× Laemmli buffer containing 50 mM Tris–HCl (pH 6.8), 2% SDS, 10% (w/v) glycerol, 0.05% bromophenol blue and 5% β-mercaptoethanol. Additionally, the cell or embryo lysates were boiled for 10 min at 90°C. The proteins were then separated by 5–18% SDS–PAGE gradient gels and transferred to PVDF membranes. Specific DPCs in cells were detected using a dot blot (Bio-Rad, Bio-Dot Apparatus 1706545) and transferred directly to a nitrocellulose membrane by vacuum aspiration. The membranes were blocked with 5% low-fat milk (Roth, T145.1) in TBST buffer (10 mM Tris–HCl (pH 7.5), 15 mM NaCl, 0.02% Tween 20) for 2 h at room temperature and then incubated with the corresponding primary antibody in 2.5% BSA in TBST overnight at 4°C. The membranes were then incubated with an HRP-labelled anti-rabbit antibody (Sigma-Aldrich. a0545, 1 : 100 000) for 1 h and washed three times for 15 min in TBST. Detection was performed according to the manufacturer's instructions for the ECL blotting substrate (Bio-Rad, 1705061) and visualized using the ChemiDoc XRS + System (Bio-Rad, 1708299).

### 2.14. Statistical analysis

ImageJ software [57] was used to quantify dot blots, western blots and morpholino-mediated silencing efficiency in zebrafish. Graphical representation of the expression data and statistical analysis was conducted using the unpaired two-sided Student's *t*-test using GraphPad Prism 8. Differences between two independent variables were considered significant when *p* < 0.05. All experiments were performed three to six times following the setup for biological replicates for RADAR isolation from cells and embryos, with means ± s.d. shown in each column.

## 3. Results

### 3.1. Comparison of zebrafish, mouse and human TDP1 proteins: phylogeny, synteny, sequence and structure

We constructed a phylogenetic tree of Tdp1 orthologues in multicellular organisms, yeast and bacteria by aligning protein sequences using the MAFFT algorithm [37] and building a phylogenetic tree using maximum-likelihood method [39]. The Tdp1 protein is very conserved in all ‘kingdoms' of life, from bacteria and yeast to plants and animals and is always present as a single orthologue (electronic supplementary material, figure S1*a*,*b* and table S1). Interestingly, the Tdp1 orthologues in invertebrates form two distinct clusters: one which is phylogenetically very close to the vertebrate cluster and the other one which is closer to the yeast and bacterial orthologues (electronic supplementary material, figure S1*a*,*b*). Human and zebrafish Tdp1 are phylogenetically very close (electronic supplementary material, figure S1*a*) and structurally very similar (figure 1*a*). We modelled the structure of zebrafish Tdp1 using the crystal structure of human TDP1 (PDB: c1nopB) [44] in the Phyre2 workspace [42]. These orthologues share a very similar structure of N and C domains with a remarkable degree of conservation (figure 1*a*). N domain (164–358 amino acid) and C domain (359–616 amino acid) form a pseudo-two-fold axis of symmetry with each domain contributing histidine, lysine and asparagine to the active site: H263, K265 and N283 in the N domain and H493, K495 and N516 in the C domain [44,58] (figure 1*a*,*c*). Upstream of the N domain, is an N terminal portion which is heavily disordered in both zebrafish and human TDP1 (1–163 and 1–144 amino acids, respectively) (electronic supplementary material, figure S1*c*). This part is highly variable among species, and its structure has not yet been solved. The amino acid sequence similarity between human and zebrafish Tdp1 is 66% (identity 55%), whereas the similarity between mouse and human is 83% (identity 77%). If we exclude the variable N terminus, similarities between orthologues are much higher: 76% between human and zebrafish (identity 66%) and 92% between human and mouse (identity 88%). After determining the phylogenetic, structural and sequence similarities between the human, mouse and zebrafish Tdp1 proteins, we analysed the gene environment of the orthologues and found that it is partially conserved. Zebrafish *tdp1* on chromosome 17 is surrounded by the upstream neighbouring gene *kcnk13a* and the downstream neighbouring genes *efcab1*1 and *foxn3* similarly as in human and mouse *TDP1* genes located on chromosomes 14 and 12, respectively (electronic supplementary material, figure S1*d*). Apart from the aforementioned neighbouring genes, the gene environment between zebrafish on one side and human and mouse *TDP1* on the other side is not conserved. By contrast, the gene environment of *TDP1* in humans and mice exhibits preserved genomic order that was presumably passed down from a common mammalian ancestor (electronic supplementary material, figure S1*d*).

### 3.2. Generation and characterization of zebrafish line lacking Tdp1 protein

The CRISPR/Cas9 system was used to generate a zebrafish strain lacking the Tdp1 protein. A gRNA targeting exon 2 induced a frameshift mutation that resulted in a premature stop codon at amino acid position 44 upstream of catalytic residues H270, K272 and N290 in the N domain (figure 1*a*,*b*). The gRNA/Cas9 complex was injected into one-cell stage embryos and fish were grown to adulthood. Identified founder fish in the F0 generation that produced germlines with a frameshift mutation that resulted in premature stop at amino acid position 44 were crossed and the F1 generation was raised. When the F1 generation reached adulthood, individuals were genotyped based on fin tissue and allele changes were sequenced. Female and male carrying described frameshift mutations (figure 1*b* and electronic supplementary material, figure S1*e*) were further crossed to produce an F2 generation deficient in Tdp1 protein (figure 1*b*). The lack of functional Tdp1 was confirmed by enzyme activity assay (figure 1*c*) and western blot using a custom-designed antibody for zebrafish Tdp1 (figure 1*d* and electronic supplementary material, figure S1*f*). For the activity assay, we used a model substrate of Tdp1, a 3′-phosphotyrosyl-DNA probe (3′pY) with a fluorescent reporter Cy-5 at the 5′ end. When Tdp1 is active, tyrosine is removed from the substrate, resulting in the oligonucleotide form (3′p) (figure 1*c*). Lysates from WT embryos (with active TDP1) were incubated with the labelled substrate and very efficient tyrosine removal was observed, whereas no reaction occurred after incubation with the lysates from *tdp1* mutant embryos demonstrating the absence of Tdp1 in the mutants (figure 1*c*). Using the custom-made Tdp1 antibody, we showed that the Tdp1 protein is indeed absent in the *tdp1* mutant line (figure 1*d*). The zebrafish antibody was intentionally designed with an epitope in a conserved protein region that overlaps between zebrafish and human TDP1 so that it could also be used to detect TDP1 in human cells. Thus, we were able to test the specificity of the new antibody by overexpressing human TDP1 in HEK293 cells (electronic supplementary material, figure S1*f*). After determining that the Tdp1 protein was indeed absent in the *tdp1* mutant line (figure 1*d*), we examined whether Tdp1 deficiency resulted in phenotype changes in embryos and adult zebrafish. There were no obvious morphological differences between WT and *tdp1* mutant embryos, nor in adult zebrafish that are now eight months old (figure 1*e* and electronic supplementary material, figure S1*g*). Future studies are needed to investigate specific phenotypes in adult Tdp1-deficient zebrafish, particularly with regard to neurodegeneration in old fish.

### 3.3. Tdp1 is highly expressed throughout embryonic development and in adult tissues

WT embryos were collected at different time points, starting at 6 h post fertilization (hpf), when most of the maternal transcriptome is degraded [59]. We show that *tdp1* is strongly expressed throughout embryonic development from 6 hpf to 5 dpf (figure 1*f*). Expression levels are highest at early stages, at 6 hpf and 1 dpf, followed by a fivefold decrease at later stages (2–5 dpf). Overall, the expression levels remain high from 6 hpf to 5 dpf (figure 1*f*). To facilitate comparison of expression levels, we set arbitrary thresholds following previous publication [60]: high expression when MNE is greater than 60 × 10^6^ (Ct values < 22), moderate when MNE is 2 × 10^6^–60 × 10^6^ (Ct = 23–26) and low when MNE is < 2 × 10^6^ (Ct > 27). *Sprtn* shows a similar expression pattern to *tdp1* at later stages, with high and stable expression levels at 2–5 dpf (figure 1*f*). *Sprtn* expression is particularly high at 6 hpf, when mRNA levels are 33-fold higher than those of *tdp1*.

In adults, *tdp1* is highly expressed in all analysed tissues, with the highest expression in testis and ovary, followed by a 3.3-fold lower expression in brain and kidney and a 14.7-fold lower expression in liver and intestine (figure 1*g*). Gender differences are not significant except in the gonads, where *tdp1* is expressed 1.3-fold more in the ovaries than in the testes. Tdp1 protein levels in zebrafish tissues corresponded to some extent to mRNA expression levels (figure 1*g*). In females, Tdp1 expression was highest in the ovaries, followed by the liver, kidney and brain, whereas the expression in the intestine was almost undetectable with the antibody used in this study (electronic supplementary material, figure S5*a*,*b*). In males, the pattern was partially similar to that in females, except for higher expression in the brain and intestine (electronic supplementary material, figure S5*c*,*d*).

To compare *tdp1* expression in zebrafish with the most commonly used animal model, the laboratory mouse, *Tdp1* expression was measured in the corresponding mouse tissues (figure 1*h*). The expression pattern is generally similar to that of the zebrafish with the highest expression in the gonads, followed by the brain, liver, kidney and intestine. However, there is a difference in the gonadal expression where *Tdp1* in mice shows higher expression in the testes than in the ovaries (although not statistically significant), whereas expression in zebrafish is 1.3-fold higher in the ovaries compared to testes (figure 1*h*).

### 3.4. Tdp1 repairs Top1- and histone H3-DPCs *in vivo*

**Figure 2.**
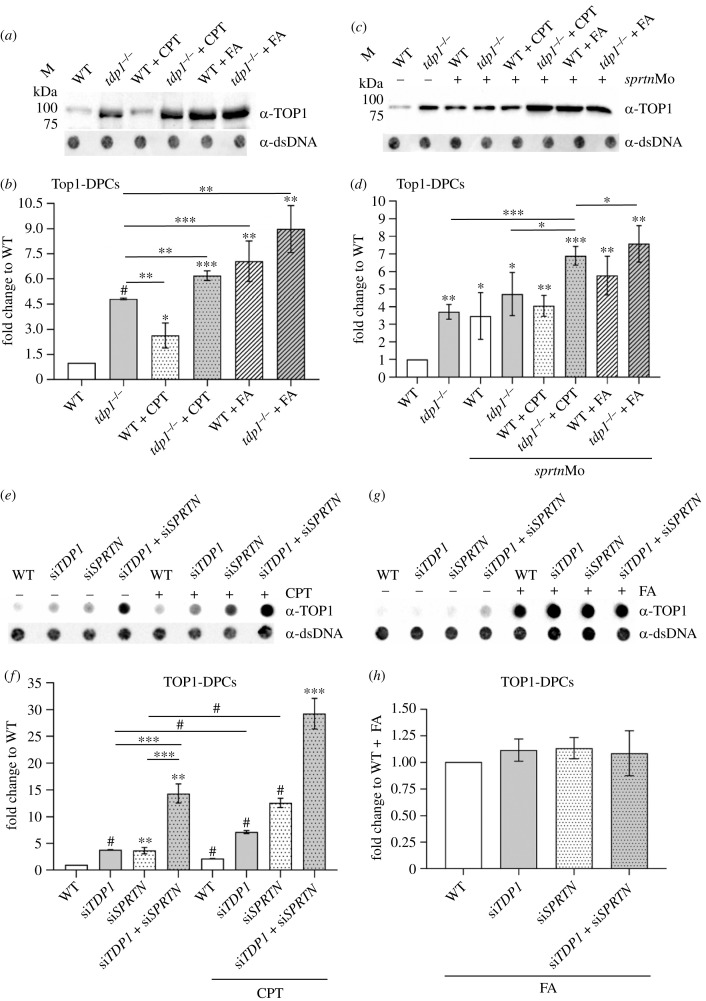
Tdp1 deficiency causes strong accumulation of endogenous and chemically induced Top1-DPCs in embryos and cells. (*a*) Western blot showing zebrafish Top1-DPCs in *tdp1* mutant embryos before and after camptothecin (CPT) (10 µM, 1 h) and formaldehyde (FA) treatment (5 mM, 30 min) (DPC equivalent of 1 µg DNA was loaded per well) and (*b*) corresponding quantification (*n* = 4). (*c*) Western blot showing zebrafish Top1-DPCs in *tdp1* mutant embryos before and after *sprtn* silencing and CPT (10 µM, 1 h) and FA treatment (5 mM, 30 min) and (*d*) corresponding quantification. (*e*) Dot blots showing human TOP1-DPCs detected with TOP1-specific antibody before and after CPT treatment of RPE1 cells (50 nM, 1 h) with corresponding DNA loading controls (DPC equivalent of 500 ng DNA was loaded per well). (*f*) Quantification of (*e*) from three different biological replicates normalized to untreated WT cells. (*g*) Dot blots showing human TOP1-DPCs detected with TOP1-specific antibody before and after FA treatment of RPE1 cells (1 mM, 20 min) with corresponding DNA loading controls (DPC equivalent of 500 ng DNA was loaded per well). (*h*) Quantification of (*g*) (*n* = 3). Results represent mean fold change ± s.d. of three different experiments. Statistically significant changes as a result from unpaired Student's *t*-test are shown as *p < 0.05, **p < 0.01, ***p < 0.001 or ^#^*p* < 0.0001.

DPC isolates were analysed for the presence of TOP1- and histone H3-DPCs by western blot and dot blot using protein-specific antibodies. Four biological replicates were used for detection of Top1- and H3-DPCs in zebrafish embryos, whereas three biological replicates were used for detection in RPE1 cells. Tdp1-deficient embryos exhibit greatly increased Top1-DPC levels under physiological conditions (4.8-fold more than WT) (figure 2*a*,*b*). In comparison, the effect of the Top1-DPC inducer, CPT (10 µM, 1 h) was weaker in WT embryos: 2.6-fold increase compared to WT (figure 2*a*,*b*). CPT further increased Top1-DPC levels in *tdp1* mutants (6.2-fold) (figure 2*a*,*b*). FA treatment (5 mM, 30 min) had a much stronger effect on Top1-DPC induction than CPT in both WT and mutant embryos: FA induced Top1-DPC levels by 7.1-fold in WT and by 9-fold in *tdp1* mutant embryos (figure 2*a*,*b*).

In RPE1 cells, the pattern of TOP1-DPC induction (figure 2*e–h*) was to some extent similar to that in embryos. TOP1-DPCs strongly accumulated in RPE1 cells after *TDP1* silencing with 3.7-fold increase compared to endogenous levels (figure 2*e*,*f* and electronic supplementary material, figure S2*c*). In comparison, CPT, a TOP1-DPC inducer, caused a 2.2-fold increase in WT cells (figure 2*e*,*f*). When cells were further challenged by the combination of TDP1 deficiency and CPT treatment, TOP1-DPC levels increased 7.1-fold compared to non-treated WT cells (figure 2*e*,*f*), confirming that TDP1 is critical for TOP1-DPC removal in human cells. In comparison, FA treatment (1 mM, 20 min) had a very strong effect on TOP1-DPC increase in WT cells, and this induction did not further increase after *TDP1* or/and *SPRTN* silencing (figure 2*g*,*h*).

**Figure 3.**
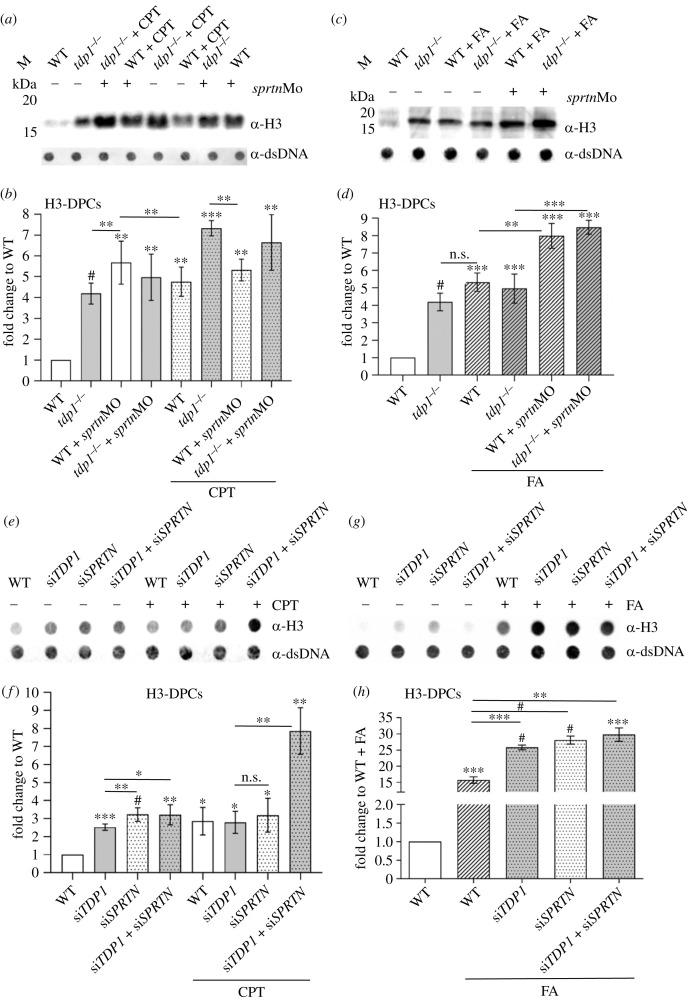
H3-DPC levels are increased *in vivo* in *tdp1* mutant fish line and in RPE1 cells with TDP1 deficiency. (*a*) Western blot showing H3-DPC levels in *tdp1* mutant embryos in combination with *sprtn* knockdown and CPT (10 µM, 1 h) treatment. Total DPCs were isolated from 2-day-old embryos, separated by SDS–PAGE (DPC equivalent of 200 ng DNA per well) and detected with H3-specific antibody. (*b*) Quantifications of H3-DPCs from four biological replicates with mean (± s.d.) fold change to endogenous H3-DPCs in WT embryos. (*c*) Western blot analysis of H3-DPCs in zebrafish embryos and (*d*) corresponding quantification after FA treatment (5 mM for 30 min) (*n* = 4). (*e*) Dot blots showing H3-DPCs after silencing *TDP1* and/or *SPRTN* before and after CPT exposure in RPE1 cells (50 nM CPT, 1 h) and DNA loading controls. Equivalent of 200 ng DNA of total DPCs was loaded per sample. (*f*) Quantification of H3-DPC analysis in RPE1 cells (*n* = 3). (*g*) Dot blots showing H3-DPCs after silencing *TDP1* and/or *SPRTN* before and after FA exposure (1 mM FA, 20 min) in RPE1 cells and DNA loading controls and (*h*) corresponding quantification (*n* = 3). Results are presented as mean ± s.d. with statistically significant changes, determined using an unpaired Student's *t*-test, indicated with *p < 0.05, **p < 0.01, ***p < 0.001 and ^#^*p* < 0.0001.

To investigate whether TDP1 is involved in the repair of DPCs other than TOP1-DPCs, we chose to examine histones as possible TDP1 substrates based on the recent *in vitro* data of Wei *et al*. [31], who showed that purified TDP1 removes crosslinked H4 and H2B at abasic sites. We chose histone H3 as a representative of the core histones because we had optimized protocols and a very sensitive antibody for its detection in the DPC isolates. In the embryos, Tdp1 deficiency caused very strong accumulation of endogenous H3-DPCs: a 4.2-fold increase compared with WT embryos (figure 3*a*,*b*). This is a very similar pattern to that observed for the canonical substrate of Tdp1, Top1-DPC, in *tdp1* mutants (4.8-fold) (figure 2*a*,*b*). CPT treatment caused a 4.7-fold increase in H3- DPCs in WT and an even greater 7.3-fold increase in *tdp1* mutants (figure 3*a*,*b*), again consistent with the pattern of Top1-DPC accumulation after CPT treatment (figure 2*a*,*b*). The FA treatment had a similarly strong effect in WT and *tdp1* mutant embryos, namely a 5.3- and 5.1-fold increase in H3-DPCs (figure 3*c*,*d*).

In RPE1 cells, *TDP1* silencing induced H3-DPC levels by 2.5-fold compared with untreated WT cells (figure 3*e*,*f*). It is important to note that CPT, although previously known to be a specific TOP1-DPC inducer, increased H3-DPCs 2.9-fold in WT cells (figure 3*e*,*f* and electronic supplementary material, figure S2*e*,*f*). H3-DPC levels were comparably affected when WT or *TDP1*-silenced cells were exposed to CPT: 2.9-fold and 2.8-fold increase, respectively (figure 3*e*,*f* and electronic supplementary material, figure S2*e*,*f*). By contrast, CPT induced many more H3-DPCs in *tdp1* mutants (7.3-fold) than in CPT-treated WT embryos (4.6-fold increase) (figure 3*a*,*b*). FA caused a remarkable 15.7-fold increase in H3-DPCs in WT cells and a 25.8-fold increase in *TDP1*-silenced cells compared with WT untreated cells (figure 3*g*,*h*).

### 3.5. SPRTN proteolysis is necessary for TOP1 and H3 DPC repair *in vivo*

To test the hypothesis that upstream proteolysis by SPRTN is required for the subsequent action of TDP1 in removing TOP1-DPCs, we quantified TOP1-DPC levels in embryos and RPE1 cells under different conditions. In WT embryos, knockdown of *sprtn* using the morpholino approach reduced *sprtn* mRNA levels by 80% (electronic supplementary material, figure S2*a*,*b*) and caused a 3.5-fold increase in Top1-DPCs levels (figure 2*c*,*d* and electronic supplementary material, figure S2*d*). In *tdp1* mutant embryos, the increase in Top1-DPCs before and after *sprtn* knockdown was 3.5- and 4.7-fold, respectively (figure 2*c*,*d* and electronic supplementary material, figure S2*d*). Surprisingly, CPT treatment (10 µM, 1 h) did not result in an additional increase in Top1-DPC levels in *sprtn-*silenced embryos (with functional Tdp1) (figure 2*c*,*d*). Compared with untreated WT embryos, CPT exposure of embryos deficient in both Tdp1 and Sprtn increased Top1-DPC levels 6.9-fold which is a significant increase compared with *tdp1* mutants and *sprtn*-silenced mutants (figure 2*c*,*d*). FA treatment (5 mM, 30 min) of *sprtn*-silenced embryos with functional Tdp1 caused a significant 5.8-fold increase in Top1-DPC levels compared with untreated WT embryos (figure 2*c*,*d*) which is an additional increase in comparison to *sprtn*-silenced embryos (3.5-fold). In *sprtn*-silenced *tdp1* mutants, exposure to FA significantly increased Top1-DPC levels compared with non-treated *sprtn*-silenced *tdp1* mutants (*p* < 0.005) (figure 2*c*,*d*).

In RPE1 cells, silencing of *SPRTN* with 65% efficiency (electronic supplementary material, figure S2*c*), resulted in a significant increase in TOP1-DPC levels (3.6-fold), which is a similar effect to silencing of *TDP1* (3.7-fold) (figure 2*e*,*f*). When both *SPRTN* and *TDP1* were silenced (electronic supplementary material, figure S2*c*), TOP1-DPCs accumulated dramatically (14.3-fold increase), suggesting that both proteins are involved in TOP1-DPC removal (figure 2*e*,*f*). The same setup after CPT exposure (50 nM, 1 h) showed a different pattern: *SPRTN* silencing caused a 12.6-fold increase, *TDP1* silencing a 7.1-fold increase, whereas double silencing additionally increased TOP1-DPC levels by 29.2-fold (figure 2*e*,*f*). FA treatment (1 mM, 20 min) dramatically increased TOP1-DPC levels to a similar extent under all conditions (figure 2*g*,*h*).

In embryos, *sprtn* knockdown had a tremendous effect on H3-DPC levels in both WT and *tdp1* mutants with 5.7- and 5.1-fold increase, respectively (figure 3*a*,*b*). CPT (10 µM, 1 h) caused a different pattern of H3-DPC induction in the same backgrounds: a 4.8-fold increase in *sprtn*-silenced embryos and a 6.6-fold increase in *sprtn*-silenced *tdp1* mutants (figure 3*a*,*b*). Both inductions were weaker than in CPT-treated mutant embryos with functional Sprtn (7.3-fold) (figure 3*a*,*b*). The levels of H3-DPCs observed in *tdp1* mutants after exposure to CPT show a significant increase compared to the endogenous levels of H3-DPCs in *tdp1* mutants (*p* < 0.0001). Moreover, this increase is even greater than the increase caused by *sprtn* silencing in *tdp1* mutants (*p* < 0.0001) (figure 3*a*,*b*). At the same time, H3-DPC levels were similarly induced after *sprtn* silencing in WT and mutant embryos before and after CPT treatment (figure 3*a*,*b*). Compared with CPT-treated WT or *tdp1* mutant embryos, knockdown of *sprtn* had no further effect on the increase in H3-DPCs (*p* < 0.05) (figure 3*a*,*b*). *Sprtn* knockdown in FA-treated embryos (5 mM, 30 min) further increased H3-DPC levels compared to embryos with functional Sprtn: 8- and 8.5-fold increase in WT and mutants, respectively, versus 5.3- and 5.1-fold increases in WT and mutants with functional Sprtn, respectively (figure 3*c*,*d*).

In RPE1 cells, SPRTN deficiency caused a very strong accumulation of H3-DPCs (3.2-fold increase) (figure 3*e*,*f* and electronic supplementary material, figure S2*e*,*f*). No additional effects on H3-DPC levels were observed when both *SPRTN* and *TDP1* were silenced. *SPRTN* silencing in untreated and in CPT-treated cells increased H3-DPCs similarly: by 3.2- and 3.2-fold, respectively (figure 3*e*,*f*). However, simultaneous silencing of *TDP1* and *SPRTN*, followed by exposure to CPT had an additive effect on H3-DPC levels resulting in a 7.9-fold increase (figure 3*e*,*f* and electronic supplementary material, S2*e*,*f*). This increase is not as strong as in TOP1-DPC levels, where CPT treatment after simultaneous silencing dramatically increased TOP1-DPCs: from 14.3-fold to 29.2-fold (figure 2*e*,*f*). When exposed to FA (1 mM, 20 min), *SPRTN*-silenced cells, as well as *SPRTN*- and *TDP1*-silenced cells exhibited a 1.8- and 1.9-fold increases in H3-DPCs, respectively, compared with FA-treated WT cells (figure 3*g*,*h*).

### 3.6. *Sprtn* silencing increases *tdp1* expression in zebrafish embryos and human cells

**Figure 4.**
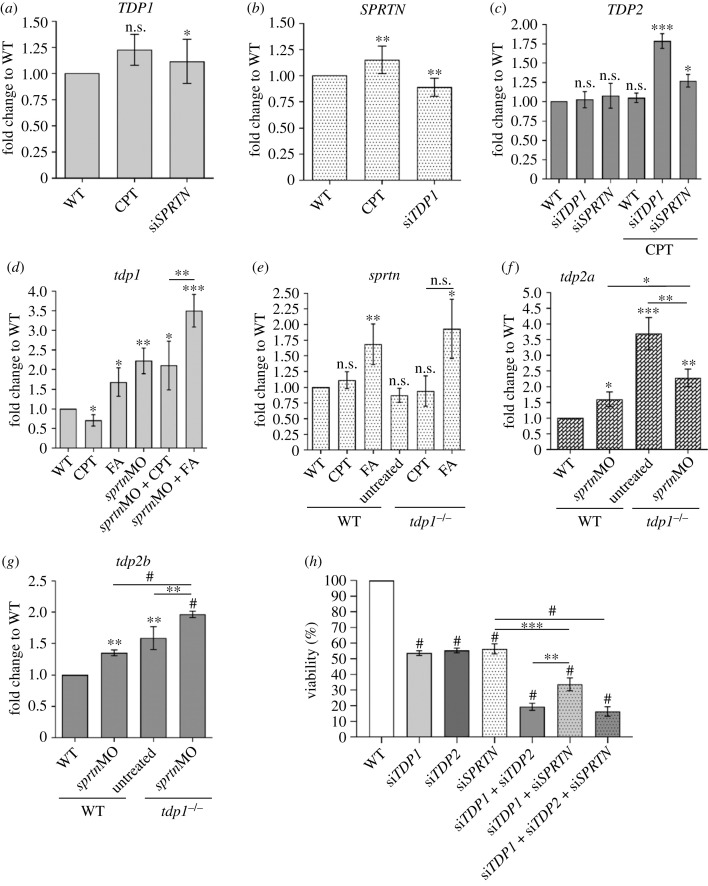
Effects of *TDP1* and *SPRTN* deficiency on *TDP1*, *SPRTN* and *TDP2* mRNA expression levels in RPE1 cells and zebrafish embryos and decrease of cell viability. (*a*) Expression levels of *TDP1* in RPE1 cells after *SPRTN* silencing and CPT exposure (50 nM, 1 h). (*b*) *SPRTN* levels decrease after *TDP1* silencing in CPT-treated RPE1 cells. (*c*) *TDP2* significantly increases after *TDP1* silencing in CPT-treated RPE1 cells. (*d*) Zebrafish *tdp1* expression levels significantly increase in embryos after *sprtn* knockdown. (*e*) *Sprtn* expression in WT and *tdp1* mutant embryos before and after CPT (10 µM, 1 h) and FA (1 mM, 20 min) treatment. (*f*) *Tdp2a* expression is significantly increased in *tdp1* mutants before and after *sprtn* silencing and in WTs after *sprtn* silencing. (*g*) *Tdp2b* expression significantly increases in *tdp1* mutants before and after *sprtn* silencing and in WTs after *sprtn* silencing. Results are presented as fold changes to WT (mean ± s.d.) from four biological replicates. (*h*) MTT viability assay after *TDP1*, *SPRTN* and *TDP2* gene silencing. All measurements were normalized to WT from three different experiments. Corresponding silencing efficiencies are shown in electronic supplementary material, figure S3 (mean ± s.d.; *n* = 3 independent experiments). Unpaired *t*-tests were performed with GraphPad Prism, with significance shown as *p < 0.05, **p < 0.01, ***p < 0.001 or ^#^*p* < 0.0001.

To investigate the interplay between TDP1 and SPRTN at the gene expression level, we quantified their mRNA levels under different conditions of gene silencing and DPC induction. In zebrafish embryos, knockdown of *sprtn* resulted in a strong 2.2-fold increase in *tdp1* expression (figure 4*d*), whereas this induction was much weaker in RPE1 cells where *SPRTN* silencing increased *TDP1* expression by 1.2-fold (figure 4*a*). Furthermore, the increase in *tdp1* mRNA levels after *sprtn* knockdown led to an increase in Tdp1 protein levels (1.25-fold compared to WT embryos) (electronic supplementary material, figure S5*e*,*f*).

Brief acute exposure of embryos to CPT (10 µM, 1 h) which strongly induced Top1-DPCs (2.8-fold) (figure 2*a*,*b*) resulted in a 29% (0.7-fold) decrease in *tdp1* expression (figure 4*d*). By contrast, a lower dose of CPT (1 h, 50 nM) in RPE1 cells, which induced TOP1-DPCs by twofold (figure 2*e*,*f*), did not significantly alter *TDP1* expression (figure 4*a*). The combination of *sprtn* silencing and CPT treatment had no further effect on *tdp1* expression compared to *sprtn*-silenced non-treated embryos (figure 4*d*). By contrast to the effects caused by CPT, treatment of WT embryos with FA (30 min, 5 mM) increased *tdp1* expression by 1.7-fold (figure 4*d*). This effect was even more pronounced in FA-treated *sprtn*-silenced embryos, in which a 3.5-fold increase in *tdp1* expression was observed (figure 4*d*). By contrast to the increase in *tdp1* mRNA levels, brief acute exposure to FA (5 mM, 30 min) had no effect on Tdp1 protein levels, probably because 30 min is too short to cause such an increase (electronic supplementary material, figure S5*e*,*f*).

The expression levels of *sprtn* in embryos were similar in WT and mutant embryos and did not change significantly after CPT exposure. However, FA increased *sprtn* expression 1.5-fold and 1.8-fold in WT and *tdp1* mutant embryos, respectively (figure 4*e*). In RPE1 cells, *TDP1* silencing caused a 10% decrease in *SPRTN* expression (figure 4*b*), whereas CPT exposure increased *SPRTN* expression by 1.2-fold (figure 4*b*). Silencing and knockdown efficiencies are shown in electronic supplementary material, figure S2*a*–*c*.

### 3.7. *Tdp2* expression increases in TDP1-deficient RPE1 cells and zebrafish embryos

In the absence of TDP1, TDP2 can participate in the Top1-mediated DNA damage in cultured DT40 cells and *in vitro* [61,62]. Therefore, we wanted to determine whether *TDP2* expression increases when TDP1 is depleted in cells and embryos. In RPE1 cells, *TDP2* expression remains the same after silencing of *TDP1* and *SPRTN* without exposure to DPC inducers. Control siRNA (siCTRL) did not affect TDP1, nor TDP2 expression levels (electronic supplementary material, figure S3*e*–*f*). However, when cells are treated with CPT (50 nM, 1 h), a TOP1-DPC inducer, *TDP2* expression increases strongly (1.8-fold) in *TDP1*-silenced cells and moderately (1.3-fold) in *SPRTN*-silenced cells (figure 4*c*). These data suggest that TDP2 may help overcome CPT-induced DNA damage in the absence of TDP1 in human cells. Because zebrafish has two *tdp2* orthologues, *tdp2a* (gene ID: 101887157) and *tdp2b* (gene ID: 553516), we performed qPCR analysis for both genes. In the absence of Tdp1, the expression of both genes increased significantly: *tdp2a* by 3.7-fold and *tdp2b* by 1.6-fold (figure 4*f*,*g*). Expression of *tdp2a* also increased after *sprtn* knockdown, by 1.6-fold in WT and 2.3-fold in *tdp1* mutants. The effect of *sprtn* silencing on *tdp2b* expression is similar to the pattern observed for *tdp2a*, with a 1.4-fold increase in WT and a 2.1-fold increase in *tdp1* mutants (figure 4*g*). By contrast, the expression pattern of the two *tdp2* orthologues in *tdp1* mutants is different: silencing of *sprtn* in mutants strongly decreased *tdp2a* expression compared with non-silenced mutants (figure 4*f*), whereas the pattern is reversed with respect to *tdp2b* expression, where silencing of *sprtn* causes an increase in *tdp2b* mRNA levels (figure 4*g*).

### 3.8. TDP1 and SPRTN deficiency affects cell viability

We observed a significant reduction in cell density after silencing of *TDP1* and *SPRTN* in RPE1 cells, so we quantified this effect using the MTT assay. Considering that TDP2 could compensate for the loss of TDP1 [61], we also investigated the effects of TDP2 deficiency in combination with the lack of TDP1 and SPRTN on cell survival. Individual silencing of *TDP1*, *TDP2* or *SPRTN* decreased cell viability by 50% (figure 4*h*). Simultaneous silencing of *TDP1* and *SPRTN* further decreased cell viability by 68% (*p* < 0.0001). Interestingly, the effect was most pronounced when both *TDP1* and *TDP2* were silenced, and viability decreased by 80%. We observed a similar effect, an 84% decrease in viability, after all three genes were silenced (figure 4*h*). The experiment was performed with three independent biological replicates, silencing efficiencies were measured for each condition and control siRNA (siCTRL) did not affect cell viability (electronic supplementary material, figure S3*d*).

### 3.9. Loss of Tdp1 leads to DPC accumulation in zebrafish embryos

**Figure 5.**
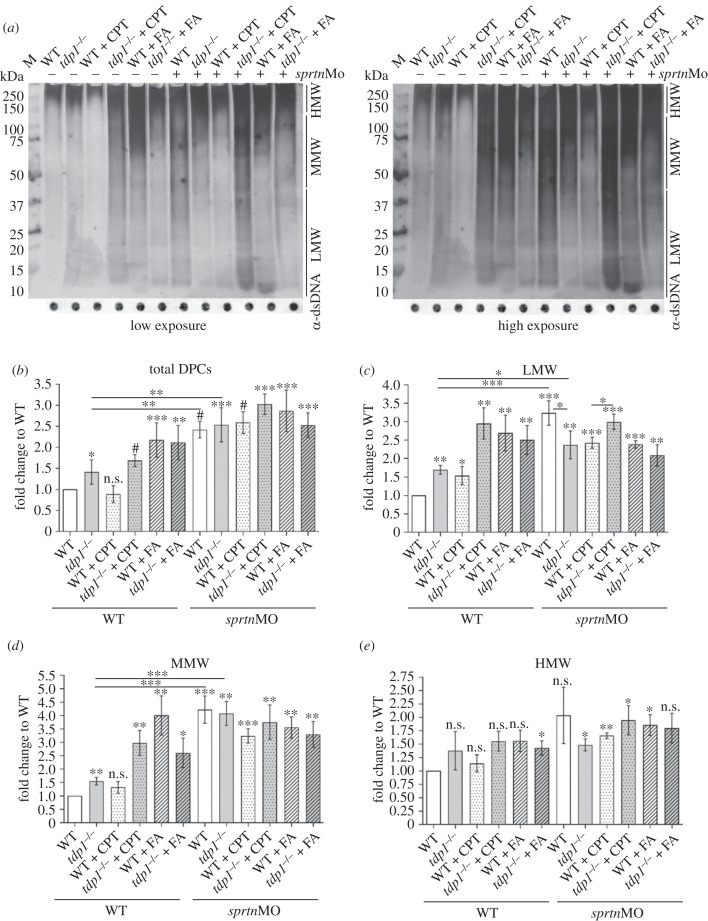
DPC analysis in Tdp1 and Sprtn deficient embryos under physiological conditions and after CPT (10 µM, 1 h) and FA (5 mM, 30 min) treatment. (*a*) DPCs were isolated from 2 dpf embryos using the RADAR assay (30 embryos per condition, *n* = 4), resolved on the SDS acrylamide gel, and stained with silver (left panel-low exposure; right panel-high exposure). Dot blots showing DNA loading controls for DPC analysis prior to benzonase treatment are shown below (DPC equivalent of 200 ng of total DNA was loaded per well). (*b*) Quantification of (*a*). Quantifications of LMW DPCs (protein size less than 40 kDa) (*c*), MMW DPCs (40–150 kDa) (*d*) and HMW (greater than 150 kDa) (*e*) from (*a*). Data represent mean fold change to WT ± s.d. (*n* = 4). Statistical significance was established using an unpaired Student's *t*-test (*p < 0.05, **p < 0.01, ***p < 0.001 and ^#^*p* < 0.0001).

*Tdp1* mutants have significantly higher endogenous DPC levels than WT embryos. The change is 1.4-fold increase and is statistically significant (figure 5*a*,*b*, *n* = 4). All experiments in embryos were repeated 4–6 times (biological replicates), because the results showed higher variability compared to experiments in RPE1 cells. CPT treatment had no effect on total DPC levels in WT embryos, but caused a significant increase (1.7-fold) in *tdp1* mutants (figure 5*a*,*b*). By contrast, the general DPC inducer, FA, caused a similar increase in total DPCs independent of Tdp1 deficiency: 2.2-fold in WTs and 2.1-fold in *tdp1* mutants (figure 5*a*,*b*). Exposure to CPT or FA had no effect on embryonic phenotype up to 2 dpf when embryos were collected for DPC analysis.

Following the analysis of the endogenous and exogenous total DPC levels in *tdp1* mutants, we investigated the interplay of Tdp1 and Sprtn in DPC removal at the *in vivo* level. Knockdown of *sprtn* resulted in a strong and significant increase in total DPC levels in both WT and *tdp1* mutant embryos (2.4-fold and 2.5-fold, respectively) (figure 5*a*,*b*). Compared with untreated WT embryos, CPT exposure increased total DPCs in *sprtn*-silenced embryos 2.6-fold in WT and 3.1-fold in *tdp1* mutants (figure 5*b*). Also, *sprtn* silencing caused a significant additive effect on total DPC increase (*p* < 0.001) in CPT-treated WT and mutant embryos (figure 5*b*). By contrast, the effect of *sprtn* silencing was not significant in either WT (*p* > 0.05) or mutant embryos (*p* > 0.05) when exposed to the general DPC inducer, FA (figure 5*b*).

Analysis of total cellular DPCs revealed important details about the functions of TDP1 and SPRTN in DPC removal and the effects of DPC inducers. However, to determine which DPCs are most affected by these changes, DPCs were divided into three subgroups: high molecular weight (HMW > 151 kDa), medium molecular weight (MMW, 40–150 kDa) and low molecular weight (LMW, 5–40 kDa). We are aware that this categorization is not ideal, but it can provide valuable additional information for the analysis of cellular DPCs compared with the analysis of total DPCs. More detailed analysis revealed that the 1.4-fold increase in total endogenous cellular DPCs in the *tdp1* mutants was indeed due to the increase in LMW and MMW DPCs, whereas HMW DPCs were not affected (figure 5*a*, high exposure). Specifically, Tdp1-deficient embryos accumulated 1.7-fold more endogenous LMW DPCs and 1.6-fold more endogenous MMW DPCs than WT embryos (figure 5*c*,*d*). CPT treatment further increased LMW DPC levels: 3.1-fold in mutants and 1.5-fold in WTs (figure 5*c*). A different pattern was observed after induction of general DPCs by FA, where levels of LMW DPCs increased 2.7- to 2.5-fold in both WT and mutant embryos (figure 5*a*,*c*). As expected, treatment with FA had strong effects on LMW DPC levels considering that most of cellular DPCs are histones [4]. Unexpectedly, LMW DPCs were also induced by CPT treatment (1.5-fold), although not as strongly as after FA treatment (figure 5*a*,*c*). When *sprtn* was knocked down, WT embryos accumulated more LMW DPCs (3.1-fold) than mutants (2.4-fold) (figure 5*a*,*c*). CPT treatment further increased LMW in mutant embryos 3.1-fold, but had no effect on LMW levels in WT embryos after knockdown of *sprtn*. LMW levels were not further affected by FA treatment when *sprtn* was silenced in WT or *tdp1*-deficient embryos (figure 5*c*).

CPT treatment of *tdp1* mutants strongly increased the levels of MMW DPCs (by 2.9-fold), in contrast to a slight statistically nonsignificant change in WT embryos (figure 5*a*,*d*). FA increased MMW DPCs by 2.6-fold in mutants and by 4.1-fold in WT embryos, showing a similar pattern of induction to LMW but with much stronger absolute changes. MMW DPCs increased 4.1-fold in both WT and *tdp1* mutants after *sprtn* knockdown (figure 5*a*,*d*). MMW DPCs in WT embryos and *tdp1* mutants were not additionally affected by CPT or FA treatment in *sprtn* knockdowns (figure 5*a*,*d*).

Tdp1 deficiency, *sprtn* knockdown, and exposure to CPT or FA had the least effect on HMW DPCs. However, some of the effects were still pronounced. HMW DPCs increased following *sprtn* knockdown by 1.5-fold in *tdp1* mutant embryos (*p* < 0.05) and by twofold in WT embryos (*p* > 0.05) (figure 5*a*,*e*). HMW DPCs increased 1.9-fold in the *tdp1* mutant and 1.6-fold in WT when *sprtn* knockdown was combined with CPT treatment. Independent of Tdp1 deficiency, knockdown of *sprtn* showed a similar induction by 1.8-fold in both the *tdp1* mutant and WT embryos which were treated with FA (figure 5*a*,*e*). Minor variations in HMW DPCs observed between the *tdp1* mutant and WT embryos with and without CPT or FA treatment were not statistically significant.

### 3.10. *TDP1* silencing causes DPC accumulation in human cells

**Figure 6.**
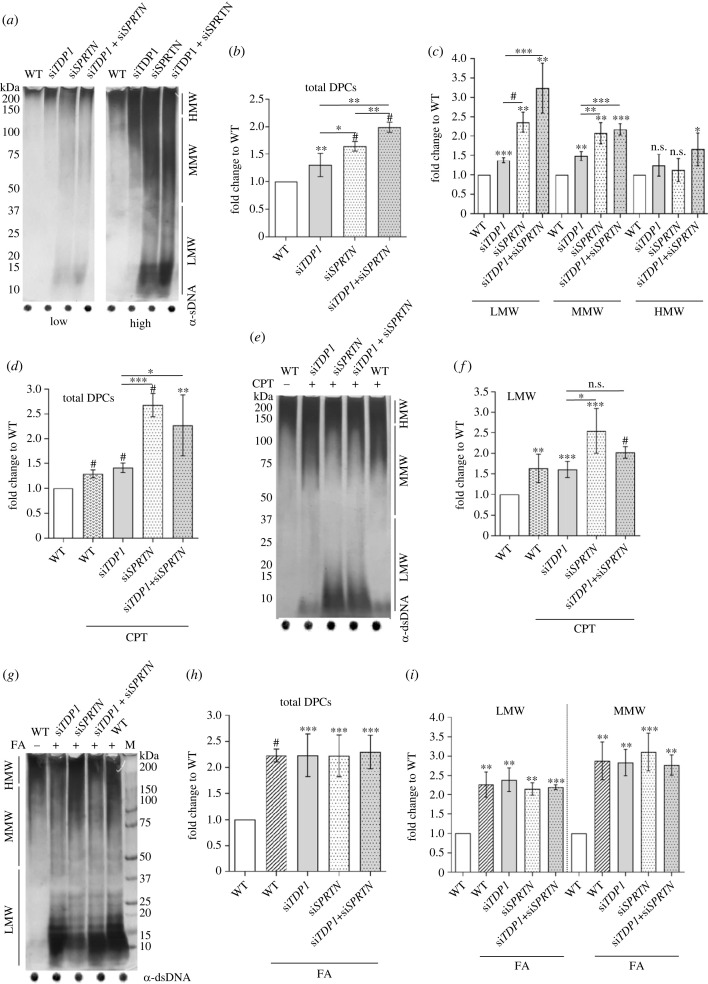
DPC analysis in RPE1 cells after *TDP1* and *SPRTN* gene silencing and after CPT (50 nM, 1 h) and FA (1 mM, 20 min) treatment. Silencing was carried out for 72 h prior to collection, and the efficiency of each condition was confirmed using qPCR (electronic supplementary material, figure S2*c*). (*a*) DPC isolates from untreated cells resolved on the SDS acrylamide gel, and stained with silver (left panel-low exposure; right panel-high exposure). Dot blots showing DNA loading controls are shown below. (*b*) Quantification of (*a*). (*c*) Quantification of LMW, MMW and HMW DPCs from (*a*) normalized to non-treated WT cells from four independent experiments (*n* = 4). (*d*) Quantification of (*e*) (*n* = 3). (*e*) DPC isolates from CPT-treated cells resolved on the SDS acrylamide gel and stained with corresponding DNA loading controls shown below. (*f*) LMW DPCs (quantification from (*d*)). (*g*) DPC isolates from FA-treated cells resolved on the SDS acrylamide gel and stained with silver with corresponding DNA loading controls. (*h*) Quantification of (*g*) (*n* = 3). (*i*) LMW and MMW DPC levels quantified from (*g*), a DPC equivalent of 200 ng total DNA was loaded per condition. All conditions were normalized to WT and statistical analysis was performed with GraphPad Prism software using an unpaired *t*-test (*p < 0.05, **p < 0.01, ***p < 0.001 or ^#^*p* < 0.0001).

DPC levels were quantified in RPE1 cells after *TDP1* and *SPRTN* silencing. Silencing of *TDP1* alone caused a small, but statistically significant increase in total DPCs (1.2-fold) (figure 6*a*,*b*), whereas silencing of *SPRTN* caused a bigger, 1.6-fold increase in DPC levels (figure 6*a*,*b*, *n* = 4). When both *TDP1* and *SPRTN* were silenced (electronic supplementary material, figure S2*c*), we observed an additive effect: a twofold increase in total cellular DPCs (figure 6*a*,*b*). Considering that SPRTN is involved in the removal of very diverse DPCs, ranging from LMW proteins such as histones to bulky HMW proteins such as topoisomerases [9], we further investigated the size distribution of the isolated DPCs. Studying the size distribution of DPCs can help us better understand which repair factors are involved in their repair and whether the function of a particular factor depends on the size of the crosslink. *TDP1* silencing increased LMW and MMW DPCs by 1.3- and 1.5-fold, respectively (figure 6*a* right panel, figure 6*c* and electronic supplementary material, figure S4*a*). The effect of the increase is not as strong as that of *SPRTN* silencing, which showed an increase of 2.6-fold in the LMW region and 1.9-fold in the MMW region (figure 6*c*). The silencing combination showed an additive effect on the increase in LMW and MMW DPCs (3.2- and 2.2-fold, respectively). The silencing combination also showed a 1.7-fold increase in HMW DPCs, in contrast to single gene silencing, where no increase was observed (figure 6*a*,*c*).

After analysing DPC accumulation in untreated cells, we quantified DPCs after exposure to CPT, a specific TOP1-DPC inducer, and after exposure to FA, a strong general DPC inducer. We used 50 nM CPT (1 h, 37°C) in serum-free medium which induces TOP1-DPCs without the occurrence of double-strand breaks [63] and 1 mM FA in ice cold serum-free medium (20 min, 37°C) which induces DPCs and probably also single- and double-strand breaks based on the data from HEK293 cells [64]. Treatment with CPT had a similar effect on DPC accumulation as did *TDP1* silencing: total DPCs increased by 1.3-fold, with the largest effect on LMW DPCs with a 1.6-fold increase (figure 6*d*–*f*). When CPT was added to *TDP1*-silenced cells, a 1.4-fold increase in total DPCs was observed (figure 6*d*), again with the largest effect on LMW with a 1.6-fold increase (figure 6*e*,*f*). CPT exposure of *SPRTN*-silenced cells caused very strong DPC accumulation (2.7-fold compared with untreated WT cells), again with the largest effect on LMW DPCs of twofold increase (figure 6*d*–*f*). By contrast to untreated cells, treatment with CPT after double silencing had no additional effect on DPC accumulation (figure 6*d*,*e*). Similar to LMW DPCs, treatment with CPT resulted in a slight 1.3-fold increase in MMW DPCs (electronic supplementary material, figure S4*b*). Interestingly, MMW DPCs in cells with silenced *TPD1*, *SPRTN* or *TDP1* and *SPRTN* were equally affected whether CPT was applied or not (electronic supplementary material, figure S4*b*). The CPT exposure had the least effect on HMW DPCs (electronic supplementary material, figure S4*b*).

By contrast to the DPC response to CPT treatment, the pattern of DPC accumulation after FA treatment was very different. FA treatment increased total DPC levels by twofold in all samples regardless of which gene was silenced (figure 6*g*,*h*). In all samples, FA treatment had the greatest impact on LMW and MMW DPCs, which increased by 2.3- and 2.8-fold on average in comparison to untreated WT cells (figure 6*i*). Proteins of HMW were least affected by FA treatment and showed no statistically significant difference in comparison to WT (electronic supplementary material, figure S4*c*).

## 4. Discussion

**Figure 7.**
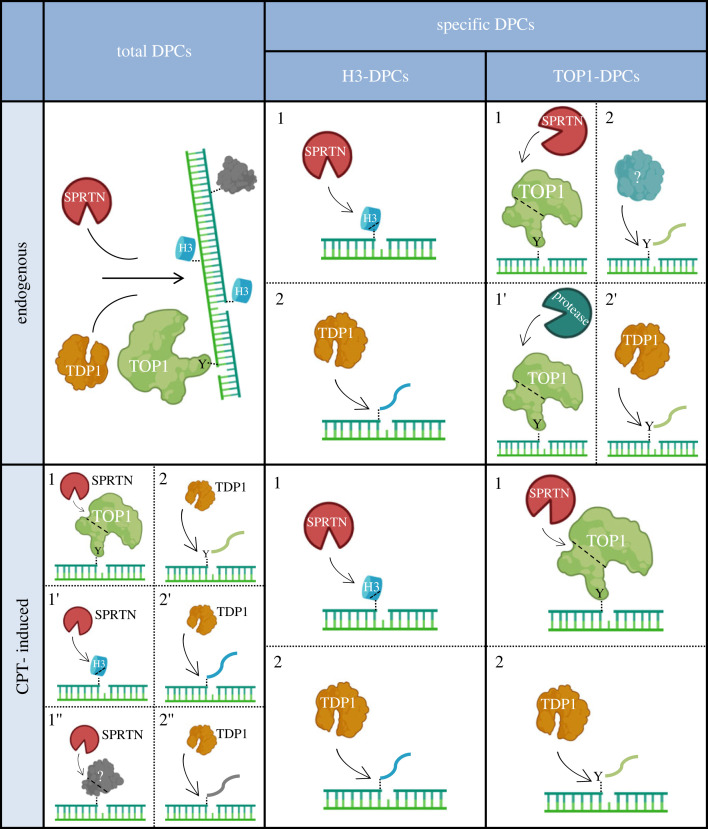
Model of coordinated action of SPRTN and TDP1 in DNA–protein crosslink repair in human cells and zebrafish model. SPRTN is a general DPC protease that cleaves a wide spectrum of crosslinked proteins, whereas TDP1 removes protein residues bound to the 3′ end of the ssDNA break. Under physiological conditions, these two proteins function independently to resolve total DPCs, including specific TOP1-DPCs. Importantly, resolution of endogenous histone-DPCs originating at abasic (AP) sites depends on SPRTN-mediated proteolysis followed by TDP1 phosphodiesterase activity, by which the crosslinked peptide residue is removed from the DNA backbone. In response to the DNA-damaging agent camptothecin, an epistatic relationship between SPRTN and TDP1 is required for the successful removal of histones and TOP1-DPCs as well as other DPCs at the 3′ ends of ssDNA breaks. The model was created with BioRender.com.

We show that TDP1 is a key factor for the repair of histone H3- and TOP1-DPCs, while SPRTN is crucial for the repair of multiple cellular DPCs including TOP1- and H3-DPCs in human cells and in zebrafish (figure 7). We further demonstrate resolution of H3-DNA crosslinks depends on upstream proteolysis by SPRTN and subsequent peptide removal by TDP1 in cells and embryos (figure 7). By contrast to H3-DPCR, where SPRTN and TDP1 work together, we show that they function in separate pathways in the repair of endogenous TOP1-DPCs (figure 7). However, after exposure of human cells to clinically relevant concentrations of CPT, SPRTN and TDP1 act epistatically in the resolution of total DPCs, histone H3- and TOP1-DPCs (figure 7).

To study DPCR *in vivo*, we established the toolbox for using the zebrafish animal model in DPC research, which includes optimization of protocols for DPC isolation and detection from embryonic tissue, generation of a Tdp1-deficient zebrafish strain, optimization of morpholino-mediated *sprtn* knockdown and development of Tdp1 antibody which recognizes zebrafish orthologue. Understanding how DPCR factors function in organisms is only possible through research in animal models, and we hope that the Tdp1-deficient zebrafish strain we have created will be a valuable resource for future studies of DPCR in specific tissues.

We analysed the degree of conservation of TDP1 between humans, mice and zebrafish and found that zebrafish is an acceptable vertebrate model for studying TDP1 function. The high degree of evolutionary conservation of one-to-one orthology across all domains of life (electronic supplementary material, figure S1*a*,*b*) and the very high structural similarity between zebrafish and human orthologues (figure 1*a*) confirm the importance of TDP1 for DNA repair throughout evolution.

High mRNA expression levels of *tdp1* and *sprtn* during embryonic development indicate that both repair factors are important in the developing embryo (figure 1*f*). This result is not surprising given rapid rate of cell division and transcription during this period and, thus, the high demand for precise DNA repair [65]. However, Tdp1*-*deficient embryos are viable and develop without any obvious phenotypic changes (figure 1*e*), suggesting compensatory mechanisms and resilience in DNA repair processes. *TDP1* is highly expressed in human testis [66] and zebrafish and mouse gonads (figure 1*g*,*h*), suggesting its protective role in germ cells. A similar mRNA tissue expression pattern of *Tdp1* in zebrafish and laboratory mouse (figure 1*g*,*h*) suggests that zebrafish would be a good model for future investigation of the tissue-specific function of TDP1 in DPCR. Comparison with human expression data is currently not possible because the mRNA expression dataset available in the Human Protein Atlas is heavily biased toward analysis of older individuals [66].

With respect to the canonical TDP1 substrate, TOP1, we found that TDP1 is crucial for the removal of TOP1-DPCs, both at the organism level and in RPE1 cells (figure 2). Consistent with previous findings in HEK293 human cells [67], the silencing of *TDP1* in RPE1 cells resulted in increased TOP1-DPC levels (figure 2*e*,*f*). By contrast, knock-out of *TDP1* in RPE1 did not lead to TOP1-DPC increase [68], suggesting adaptive mechanisms in permanent knock-out as opposed to temporary gene silencing. Given the importance of TOP1 inhibition in cancer therapy [13,69], the role of TDP1 in TOP1-DPCR has been extensively investigated *in vitro* and in cell cultures [32,70–72], but data on the vertebrate models are sparse [47,73,74]. Using zebrafish model, we show that Tdp1 deficiency leads to a significant 4.2-fold increase in endogenous Top1-DPCs (figure 2*a*,*b*), demonstrating that TDP1 is a crucial repair factor for the resolution of TOP1-DPCs. Previous study by Zaksauskaite *et al*. [47] did not report difference in Top1-DPC levels between wild-type and Tdp1-deficient zebrafish embryos. The discrepancy is likely due to the different approaches to DPC isolation, as the RADAR assay used in this study is more specific and sensitive than the CsCl fractionation method used in the previous study [4,49,75]. Expression analysis of *tdp2* showed a significant increase in *tdp2* when *tdp1* was absent in embryos suggesting that TDP2 may compensate for loss of TDP1 *in vivo*, supporting previous observations from cell culture and *in vitro* experiments [61,62]. Detailed insights into TOP1-DPCR at the organism level are important for biomedicine, because TOP1-DPC inducers, the CPT derivatives irinotecan and topotecan, are used to treat various cancers including ovarian and colon cancers, small-cell lung cancer, central nervous system tumours and sarcomas [69]. Combination therapies with TOP1 and TDP1 inhibitors could significantly improve current clinical protocols, and thus it is essential to know how TDP1 functions in the repair of DPCs at the organism level.

It is also very important to understand how histone-DPCs are repaired because they are very abundant under physiological conditions. More than 10 000 abasic sites are generated daily [76], and about 10% of these lead to the formation of DPCs, most of which are histone-DPCs [4]. This suggests that hundreds, possibly even thousands of histones are crosslinked to abasic sites in each individual cell every day [77]. Induction of histone-DPCs may be a promising new avenue to explore in the treatment of cancer. Our discovery that TDP1 is a critical factor in histone-DPCR (figure 3) further highlights TDP1 as an important drug target. Our results support recent observations of Wei *et al*. [31], who showed that TDP1 can remove histone H2B and H4 from AP sites *in vitro*, and provide evidence for a novel, TDP1-dependent repair pathway for histone-DPC resolution *in vivo*. We also observed that loss of Tdp1 in zebrafish embryos and RPE1 cells results in a significant increase in cellular DPCs that cannot be attributed solely to the increase in H3- and TOP1-DPCs (figures 5 and 6), suggesting that TDP1 has multiple DPC substrates. On the other hand, the increase in LMW DPCs in *tdp1* mutant embryos and TDP1-deficient RPE1 cells (figures 5*c* and 6*c*) is most likely due to the increase in histone-DPCs, considering that endogenous H3-DPCs accumulate strongly in Tdp1-deficient embryos and human cells (figure 3). The function of TDP1 in the repair of cellular DPCs and histone-DPCs has not been previously investigated and our results should be considered in the development of TDP1 inhibitors for cancer therapy [15].

Regarding the role of SPRTN protease in DPCR, we show that SPRTN is a crucial protease for the resolution of multiple cellular DPCs *in vivo*, and in particular for the removal of low- and medium-molecular weight DPCs (figures 5 and 6). By analysing DPCs in SPRTN-deficient embryos, we provide the first evidence of how DPC levels are affected in an organism. Compared with embryos, the effect of SPRTN deficiency was somewhat weaker in RPE1 cells. We have shown that SPRTN is critical for TOP1-DPCR at the organism level (figure 2*c*,*d*), supporting previous studies in cell culture that showed an increase in TOP1-DPC levels after SPRTN silencing [5,7,9] and that SPRTN proteolyzes TOP1 *in vitro* [9]. Our results also show that SPRTN plays an important role in resolving H3-DPCs *in vivo*, as knockdown of *sprtn* in zebrafish resulted in a 5.7-fold increase in H3-DPC levels (figure 3*a*,*b*), supporting *in vitro* data characterizing H3 as a substrate of SPRTN [9]. Therefore, our study finally demonstrates the crucial role of SPRTN in resolving DPCs at the organism level and highlights SPRTN as a promising chemotherapeutic target.

We characterized the interplay of TDP1 and SPRTN in DPC resolution under physiological conditions. SPRTN and TDP1 are known to play distinct roles in DPCR. SPRTN is required for initiating repair of many crosslinked proteins [3], whereas TDP1 has previously been specifically associated with repair of TOP1-DPCs [78]. The function of TDP1 in the repair of DPCs other than TOP1-DPCs has not been previously studied *in vivo*. Indeed, our results in RPE1 cells suggest a non-epistatic relationship between these two proteins in the repair of total cellular DPCs (Figures 6*a*,*b* and 7) and in the repair of endogenous TOP1-DPCs (Figures 2*e*,*f* and 7). We observed a similar pattern in embryos, but the changes were not statistically significant in this case because of higher variability between experiments (figure 2*c*,*d*). Since it is known that TDP1 cannot process TOP1-DPCs alone [32], we hypothesize that another protease is involved in the upstream proteolysis of endogenous TOP1-DPCs (figure 7). Possible candidates include DDI1, DDI2, FAM111A and the proteasome [17,19,21,79]. Unlike in endogenous TOP1-DPCR, we show that SPRTN and TDP1 work together in the resolution of endogenous H3-DPCs in zebrafish and in human cells (figure 3*a*,*b*,*e*,*f*), suggesting that SPRTN is the main protease acting upstream of TDP1-mediated peptide removal in the resolution of histone-DPCs at AP sites (figure 7). Our results are the first to show that SPRTN proteolysis is required for histone-DPC resolution *in vivo*. It is worth noting that *in vitro*, TDP1 can remove H2B and H4 crosslinks without the requirement of upstream proteolysis [31]. Known discrepancies between *in vitro* and *in vivo* data further emphasize the urgent need for the experimental data from the animal models. The interplay between SPRTN and TDP1 is also evident at the level of mRNA and protein expression, where silencing of *SPRTN* increases *TDP1* expression in embryos and cells (figure 4*a*,*d*). Both proteins are critical for normal cell function, as RPE1 cells with silenced *TDP1* and *SPRTN* exhibit a 68% reduction in viability (figure 4*h*). We suggest that this phenotype is the result of impaired DPCR resulting from the absence of TDP1 and SPRTN (figures 2, 3, 5 and 6). It is known that *SPRTN*-silenced human cells exit S phase with abnormal replication intermediates [64] and *TDP1*-silenced cells exhibit an increase in ssDNA and dsDNA breaks [67].

By contrast to physiological conditions, after exposure to CPT, when TOP1-DPCs are induced, we show that SPRTN and TDP1 act epistatically in resolving total DPCs in RPE1 cells (figures 6*a*,*b*,*d*,*e* and 7). Our results are consistent with data from cell survival assays showing that simultaneous depletion of TDP1 and SPRTN in yeast [5,80] and HeLa cells [9] to a similar extent as depletion of either component alone leads to hypersensitivity to treatment with CPT, suggesting that SPRTN and TDP1 function in the same pathway for the repair of CPT-induced TOP1-DPCs. It is important to note that DPCR factors may behave differently under physiological conditions and under stress, when DPC load exceeds certain thresholds. In summary, our results suggest that repair of CPT-induced TOP1-DPCs relies on the SPRTN-TDP1 axis, in contrast to repair of endogenous TOP1-DPCs, in which TDP1 and SPRTN function in separate pathways (figure 7). It is important to note that CPT also induces H3-DPCs in human cells and in zebrafish embryos (figure 3; electronic supplementary material, figure S2*e*,*f*), demonstrating for the first time that CPT is not specific for TOP1-DPC induction, as previously thought [81]. This effect is likely indirect, considering that CPT has been characterized as an agent that directly crosslinks TOP1 [69]. It is conceivable that the increased requirement for TOP1-DPCR after CPT exposure leads to decreased recruitment of TDP1 to H3-DPC lesions, which are endogenously very common, ultimately leading to an increase in H3-DPCs. However, this remains to be investigated in future studies. FA is a potent crosslinker of various cellular proteins ranging in size from 10 kDa to over 200 kDa [5,80,82]. Our results show that the interplay of SPRTN and TDP1 in DPCR is altered when cells and embryos are exposed to high acute doses of FA, compared with physiological conditions in which only endogenous DPCs are present. SPRTN and TDP1 act together (in epistasis) in the repair of FA-induced total cellular DPCs (figures 5 and 6*g*,*h*,*i*) and in the repair of FA-induced H3- and TOP1-DPCs (figures 2 and 3). Considering that large amounts of DPCs accumulate under these conditions, we hypothesize that SPRTN is fully activated and performs upstream proteolysis of many crosslinked proteins given its pleiotropic nature [9], and that TDP1 is crucial for the resolution of H3- and TOP1-DPCs and other crosslinked protein residues at AP sites.

In summary, we have performed a comprehensive analysis of the role of TDP1 and SPRTN in the resolution of DPCs in zebrafish and human cells. Our results reveal the interplay of these repair factors in the resolution of cellular DPCs, H3- and TOP1-DPCs and introduce a novel TDP1-mediated repair pathway for histone-DPCs that highlights the epistatic relationship between upstream SPRTN proteolysis and downstream TDP1-mediated 3′ DNA end processing (figure 7). Furthermore, we demonstrate the essential role of both proteins in the resolution of total cellular DPCs and TOP1-DPCs in human cells and in the animal model. Our results provide new insights into the complex DPCR pathways and their implications for human disease and cancer treatments. Further research in this area will advance our understanding of DPCR factors and their potential therapeutic applications. It is important to point out that mechanistic, *in vitro* studies are essential for understanding DPCR processes, but that research in animal models is essential for understanding and contextualizing the interplay of the various repair factors in the whole organism which is a prerequisite for translating the acquired knowledge for understanding and treating human diseases. The mechanism of action of TDP1 and SPRTN has been previously studied in detail, but how these repair factors function together in cells and tissues in DPCR was previously unknown.

## Data Availability

The data are provided in electronic supplementary material [83].
